# Supplementary material for: Hybrid purity identification using EST-SSR markers and heterosis analysis of quantitative traits of Russian wildrye
Source: PeerJ. 2022 Nov 30;10:e14442. doi: 10.7717/peerj.14442 (PMC9744169; doi:10.7717/peerj.14442)
Supplement: Supplemental Information 1 — NPGS, U.S., U.S. National Plant Germplasm System. [file peerj-10-14442-s001.docx]

| **Accession** | **Variety ( material )** | **Origin** | **Germplasm preservation institutions** |
| --- | --- | --- | --- |
|  |  |  |  |
| PI 272136 | - | Alma-Asa, kazakhstan | NPGS, U.S. |
| PI 406468 | BOZOISKI | Former Soviet Union | NPGS, U.S. |
| PI 476299 | VINALL | United States | NPGS, U.S. |
| PI 502573 | AR-163 | Former Soviet Union | NPGS, U.S. |
| PI 502576 | K 37772 | Russian Federation | NPGS, U.S. |
| PI 502577 | K 40175 | Russian Federation | NPGS, U.S. |
| PI 531826 | D-3139 | China | NPGS, U.S. |
| PI 565044 | DJ-3890 | Russian Federation | NPGS, U.S. |
| PI 565060 | AJC-534 | Russian Federation | NPGS, U.S. |
| PI 565051 | DJ-4154 | Russian Federation | NPGS, U.S. |
| PI 565052 | DJ-4155 | Russian Federation | NPGS, U.S. |
| PI 595135 | X 93031 | Xin jiang, China | NPGS, U.S. |
| PI 598610 | X 93031 | Xin jiang, China | NPGS, U.S. |
| PI 598611 | VIR U-0134932 | Kazakhstan | NPGS, U.S. |
| PI 619483 | 96N-331 | Mongolia | NPGS, U.S. |
| PI 619487 | 96N-300 | Mongolia | NPGS, U.S. |
| PI 549118 | ‘BOZOISKY SELECT’ | Utah, U.S. | NPGS, U.S. |
| CF 005038 | XJ-ALT | Xin jiang, China | National Medium term Gene Bank of Forage Germplasm, China |
