# Supplementary material for: Hybrid purity identification using EST-SSR markers and heterosis analysis of quantitative traits of Russian wildrye
Source: PeerJ. 2022 Nov 30;10:e14442. doi: 10.7717/peerj.14442 (PMC9744169; doi:10.7717/peerj.14442)
Supplement: Supplemental Information 2 — p ≥ 0.05 = At 0.05 level, this group of data is significant from the normal distribution population [file peerj-10-14442-s002.docx]

| **Items (P value)** | **Year** | | |
| --- | --- | --- | --- |
|  | **2019** | **2020** | **2021** |
| Tiller height (cm) | 0.198 | 0.062 | 0.100 |
| Second leaf length (cm) | 0.226 | 1.000 | 0.428 |
| Second leaf width (cm) | 0.040 | 0.251 | 0.856 |
| Reproductive tiller number | 0.155 | 0.013 | 0.314 |
| Nutritional tiller number | 0.157 | 0.069 | 0.100 |
| Seed number per plant (/1000) | 0.002 | 0.001 | 0.002 |
| Spike length (cm) | 1.000 | 0.917 | 0.942 |
| Spike width (cm) | 0.581 | 0.157 | 1.000 |
| Spikelet number per spike | 0.935 | 0.415 | 0.451 |
| Seed number per spike | 0.174 | 1.000 | 0.290 |
| Seed weight per spike (g) | 0.443 | 0.677 | 0.449 |
| Thousand kernel weight (g) | 0.386 | 0.735 | 0.371 |
