# Supplementary material for: Hybrid purity identification using EST-SSR markers and heterosis analysis of quantitative traits of Russian wildrye
Source: PeerJ. 2022 Nov 30;10:e14442. doi: 10.7717/peerj.14442 (PMC9744169; doi:10.7717/peerj.14442)
Supplement: Supplemental Information 3 [file peerj-10-14442-s003.docx]

| **SSR Type** | **Number** | |
| --- | --- | --- |
|  | **ALL Unigenes with SSRs** | **DEGs with SSRs** |
| c | 402 | 11 |
| p1 | 3748 | 182 |
| p2 | 1584 | 73 |
| p3 | 3358 | 128 |
| p4 | 178 | 5 |
| p5 | 21 | 1 |
| p6 | 9 | 0 |
| Total | 9300 | 400 |
