# Supplementary figures and images for: Hybrid purity identification using EST-SSR markers and heterosis analysis of quantitative traits of Russian wildrye
Source: PeerJ. 2022 Nov 30;10:e14442. doi: 10.7717/peerj.14442 (PMC9744169; doi:10.7717/peerj.14442)

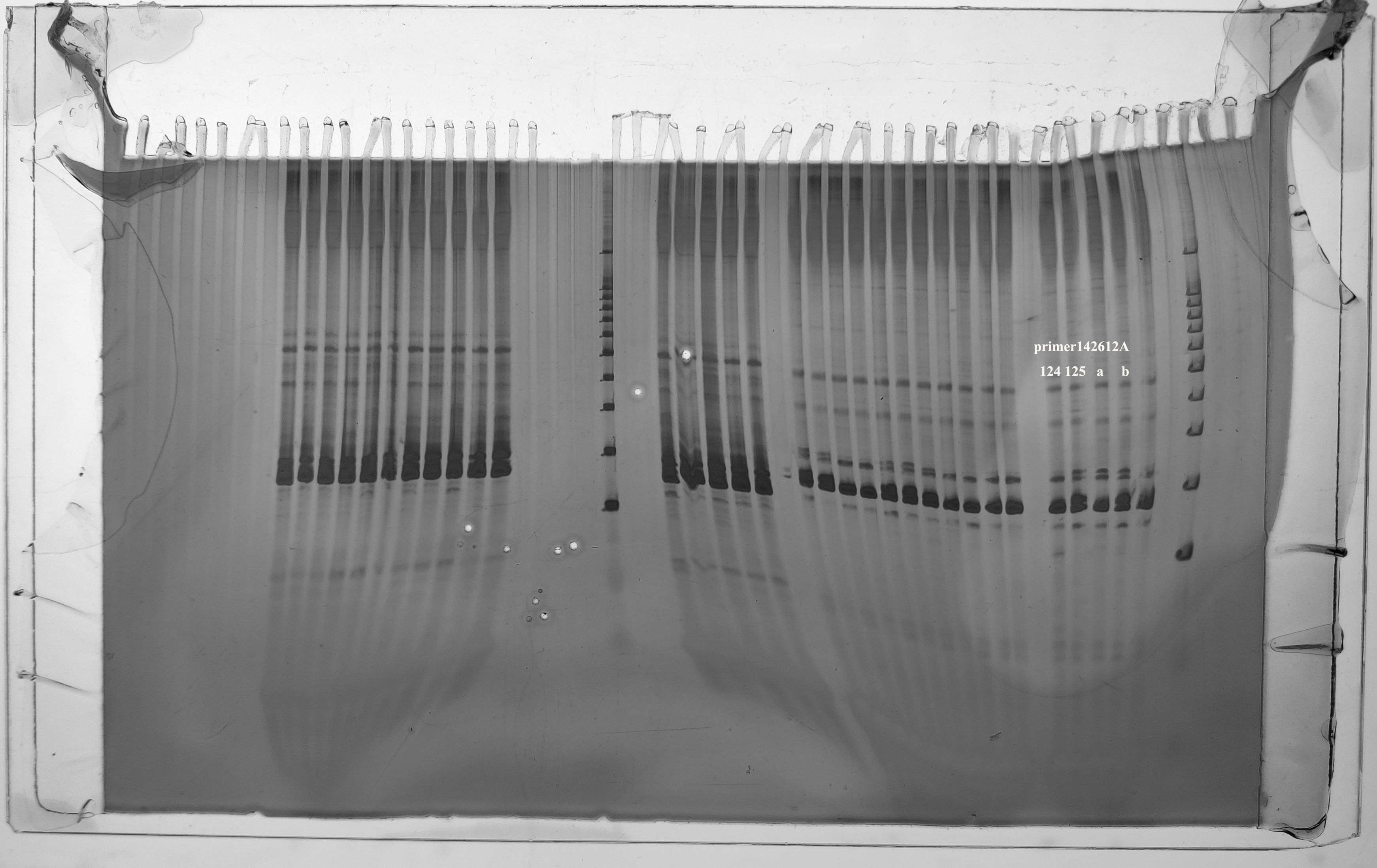

Supplement: Supplemental Information 6 — a = the male parent of BC1 population; b = the female parent of BC1 population; 124 = the female parent of BC1 population; 125 = the female parent of BC1 population [file peerj-10-14442-s006.zip › primer screening/142612A.jpg]

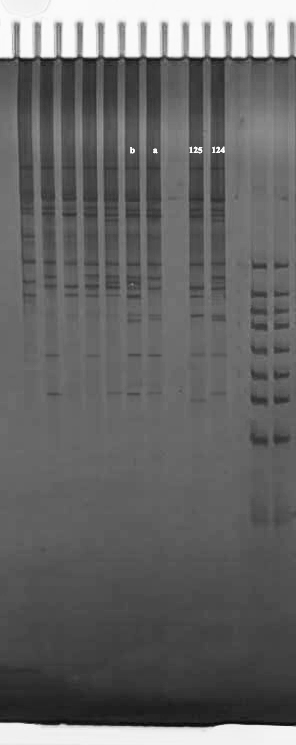

Supplement: Supplemental Information 6 — a = the male parent of BC1 population; b = the female parent of BC1 population; 124 = the female parent of BC1 population; 125 = the female parent of BC1 population [file peerj-10-14442-s006.zip › primer screening/28553.jpg]

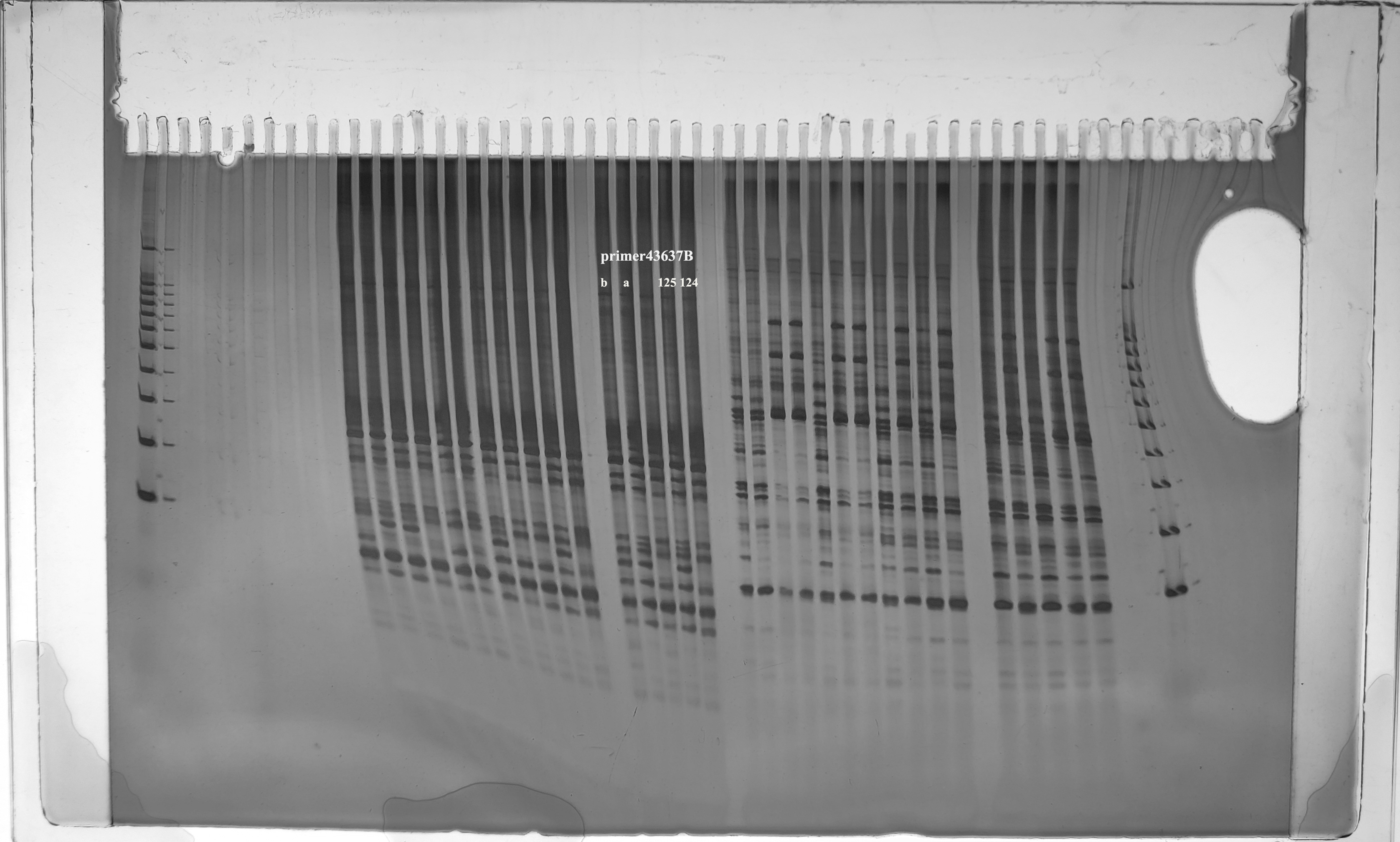

Supplement: Supplemental Information 6 — a = the male parent of BC1 population; b = the female parent of BC1 population; 124 = the female parent of BC1 population; 125 = the female parent of BC1 population [file peerj-10-14442-s006.zip › primer screening/43637B.jpg]

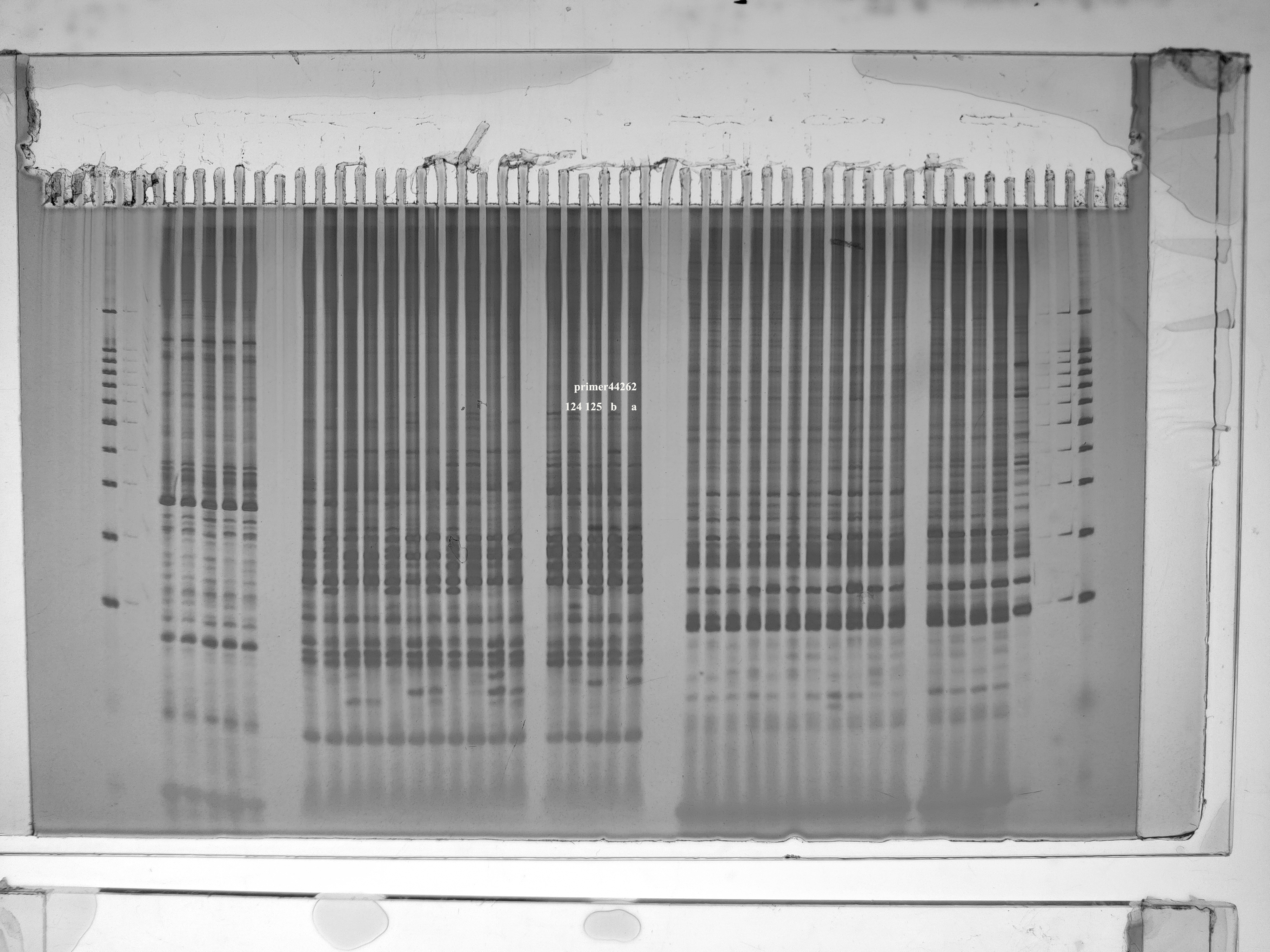

Supplement: Supplemental Information 6 — a = the male parent of BC1 population; b = the female parent of BC1 population; 124 = the female parent of BC1 population; 125 = the female parent of BC1 population [file peerj-10-14442-s006.zip › primer screening/44262.jpg]

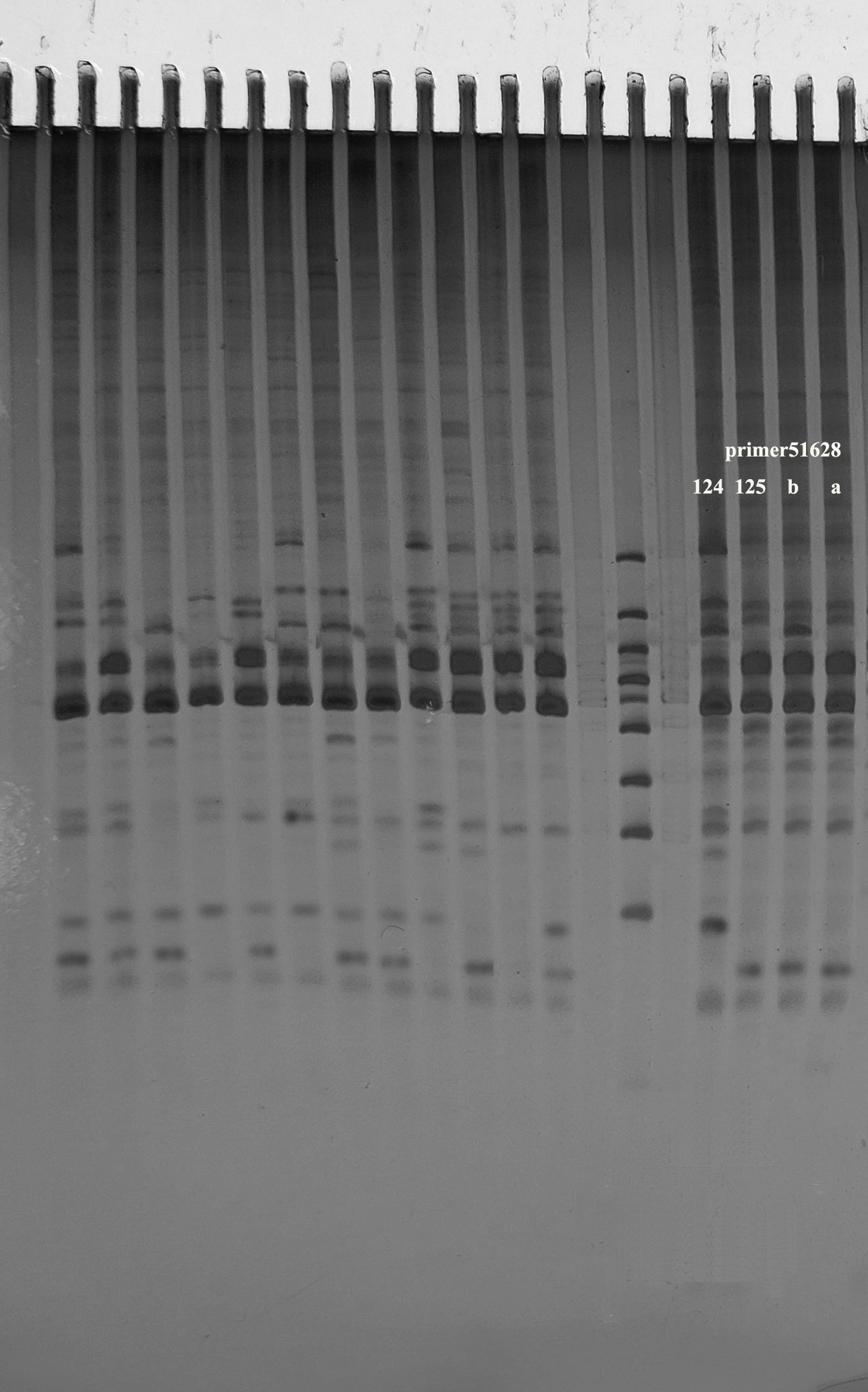

Supplement: Supplemental Information 6 — a = the male parent of BC1 population; b = the female parent of BC1 population; 124 = the female parent of BC1 population; 125 = the female parent of BC1 population [file peerj-10-14442-s006.zip › primer screening/51628.jpg]

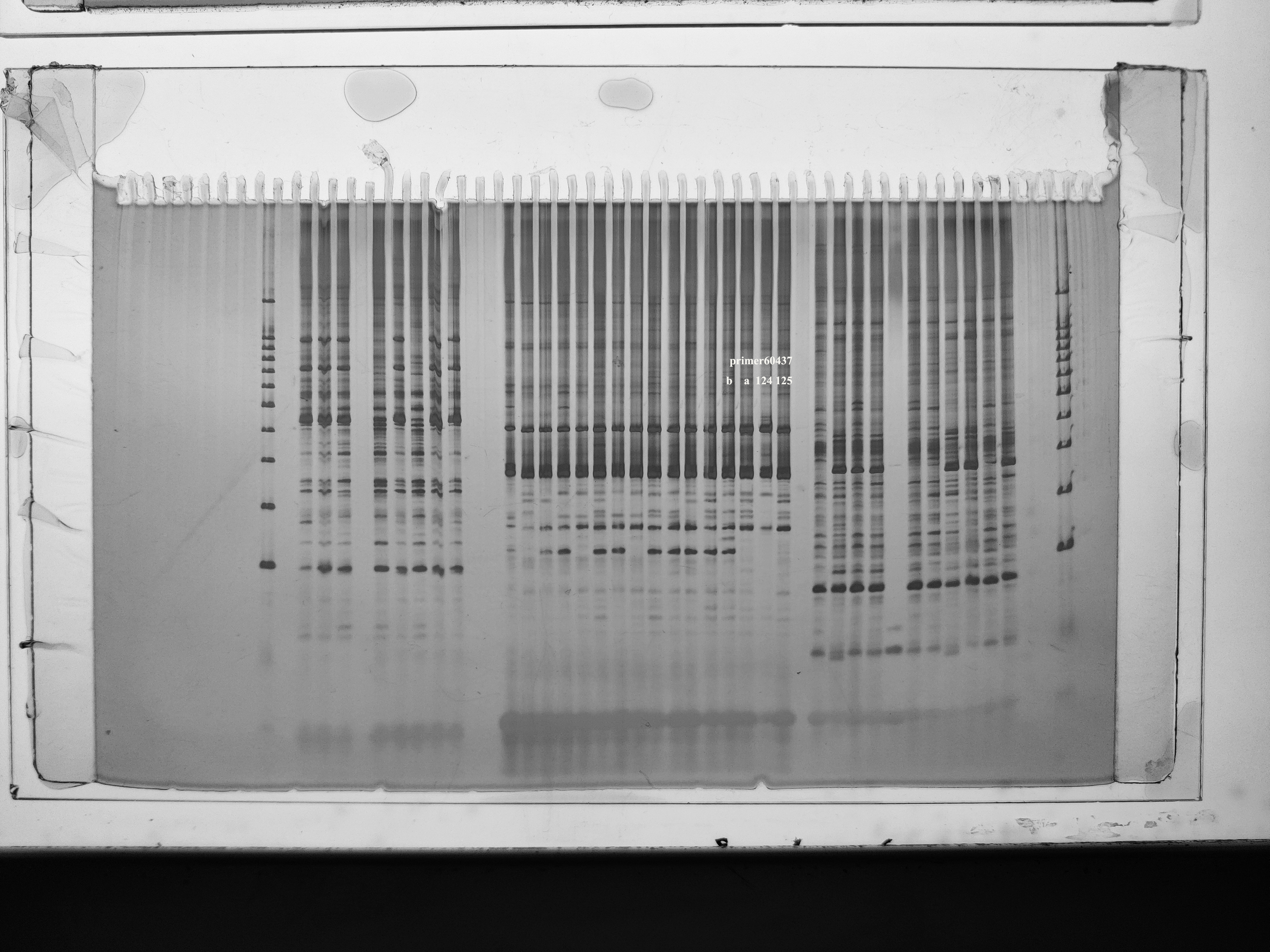

Supplement: Supplemental Information 6 — a = the male parent of BC1 population; b = the female parent of BC1 population; 124 = the female parent of BC1 population; 125 = the female parent of BC1 population [file peerj-10-14442-s006.zip › primer screening/60437.jpg]

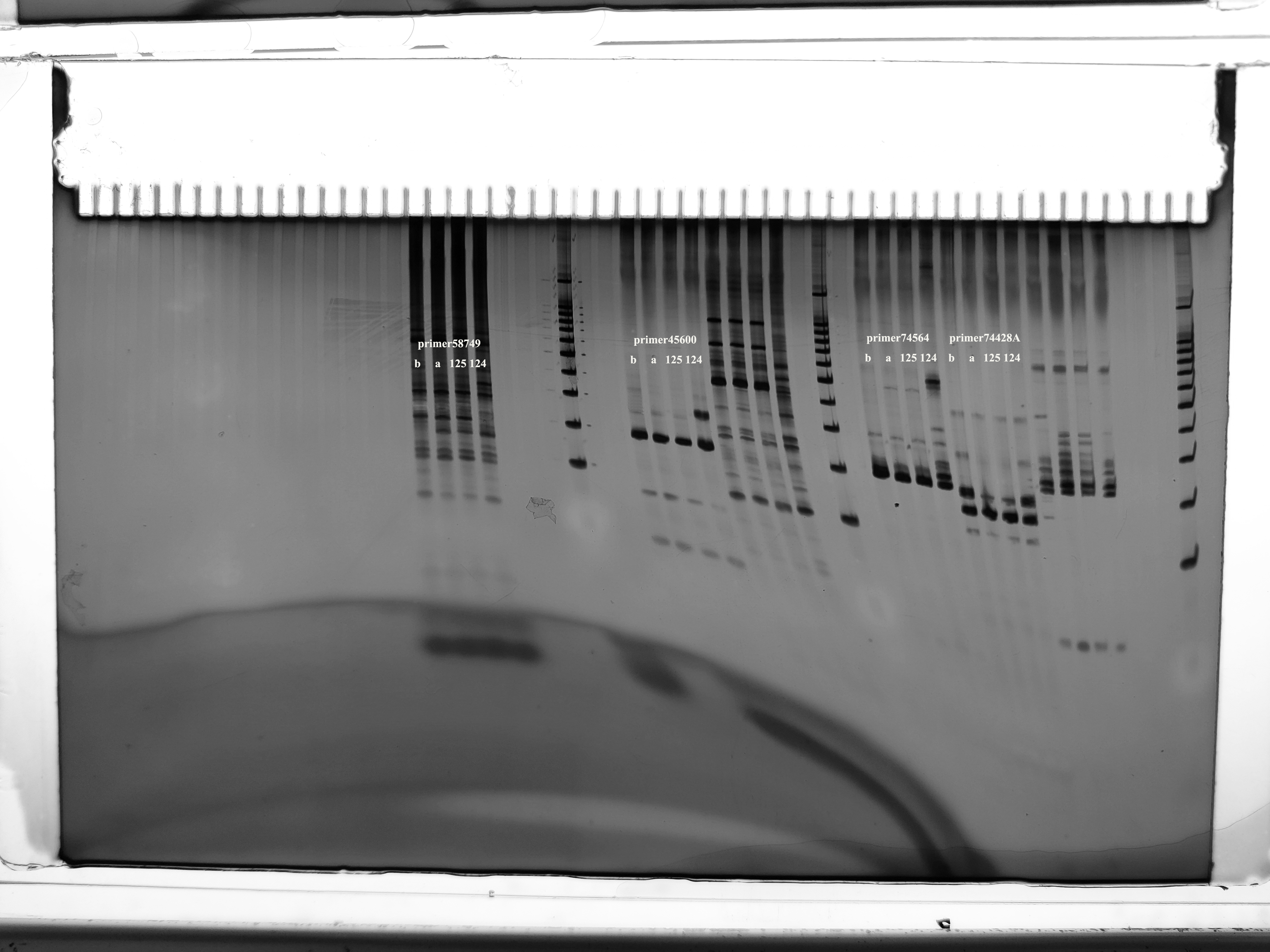

Supplement: Supplemental Information 6 — a = the male parent of BC1 population; b = the female parent of BC1 population; 124 = the female parent of BC1 population; 125 = the female parent of BC1 population [file peerj-10-14442-s006.zip › primer screening/74428A, 74564, 45600.jpg]

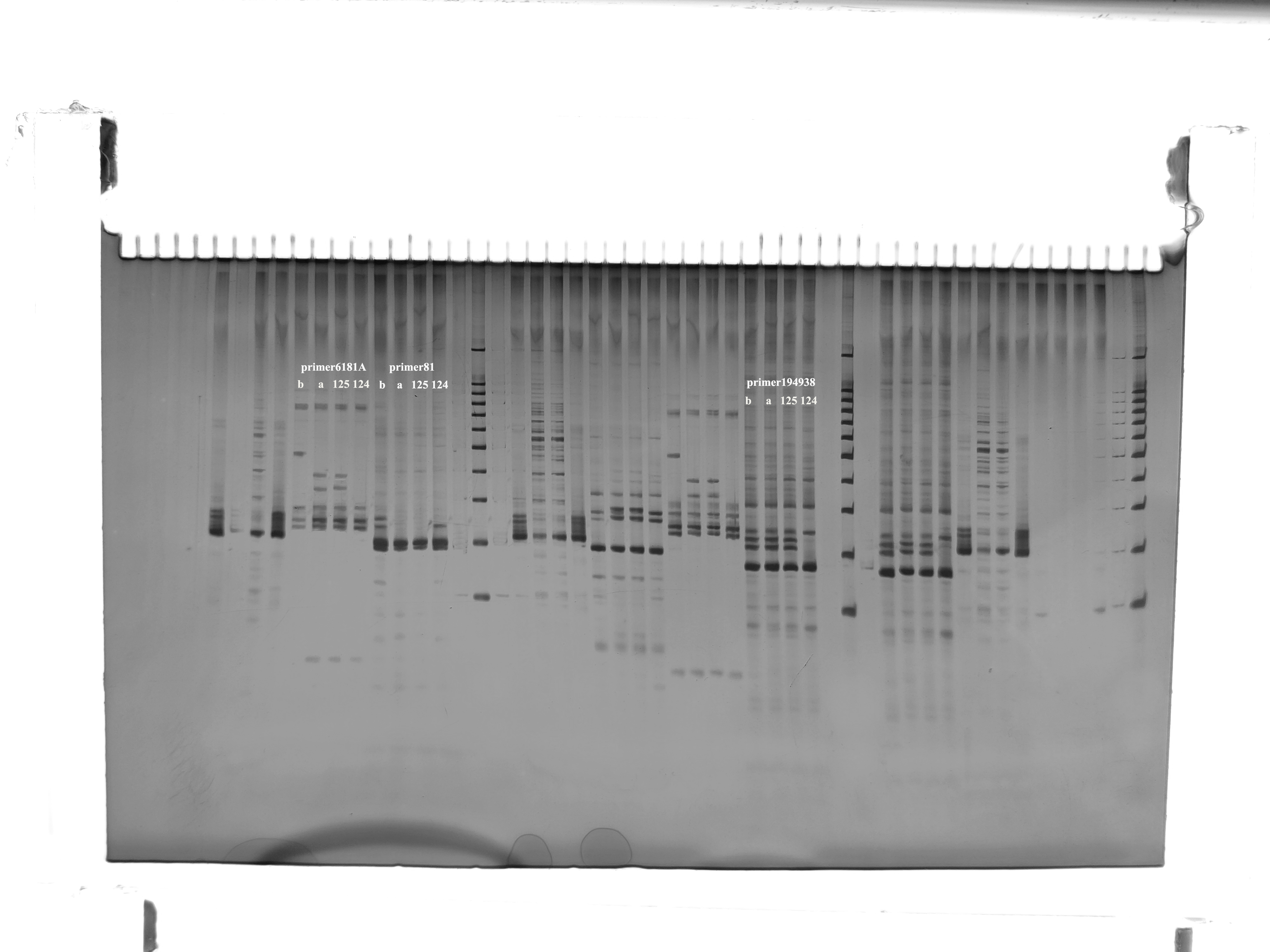

Supplement: Supplemental Information 6 — a = the male parent of BC1 population; b = the female parent of BC1 population; 124 = the female parent of BC1 population; 125 = the female parent of BC1 population [file peerj-10-14442-s006.zip › primer screening/81, 6181A, 194938.jpg]

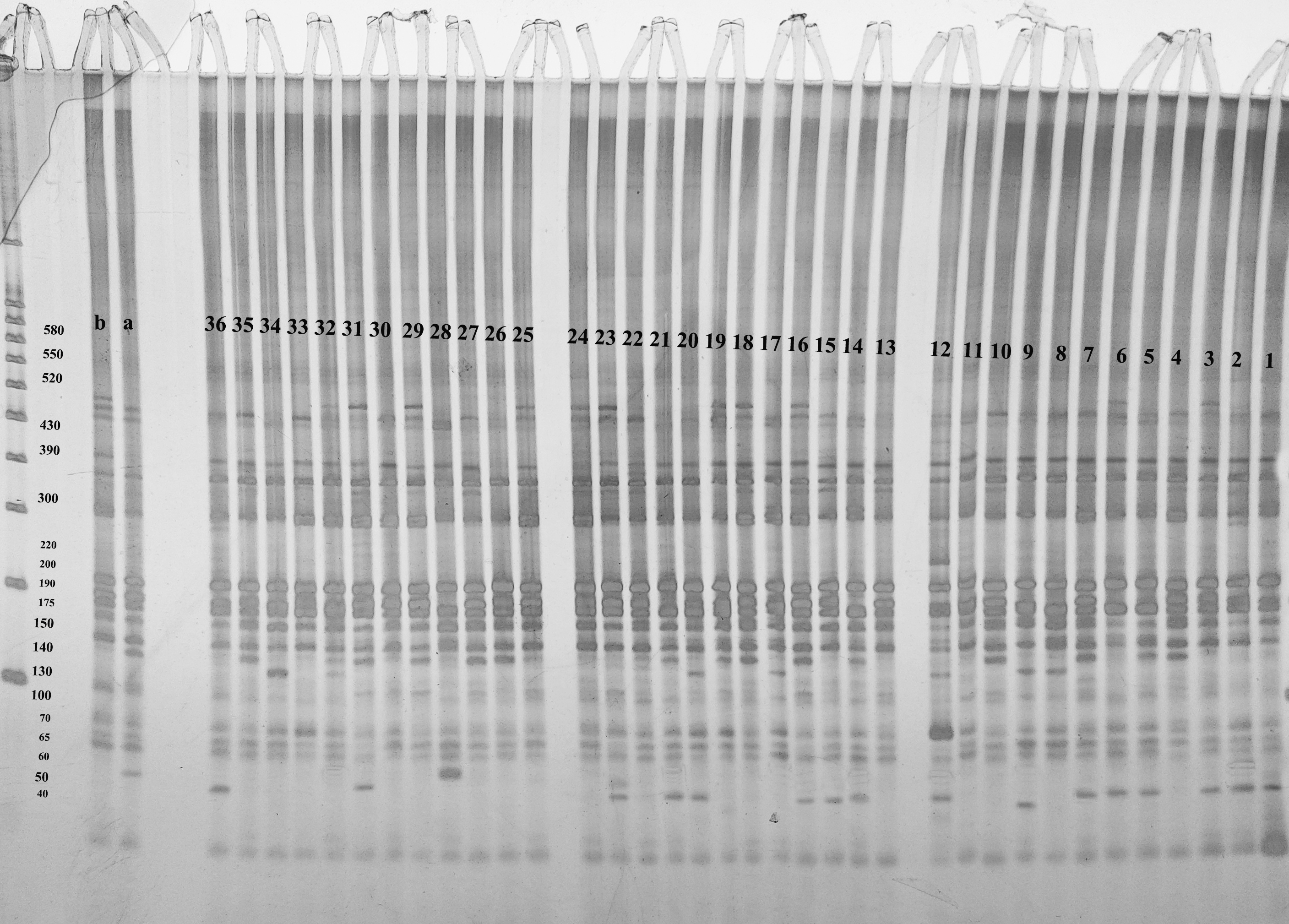

Supplement: Supplemental Information 8 — The compressed file name is the primer name, the electrophoresis lane number is expressed by the subfile name, and the lane number is marked above the electrophoresis lane in the picture. [file peerj-10-14442-s008.zip › BC1/44262/1-36+a,b(44262).jpg]

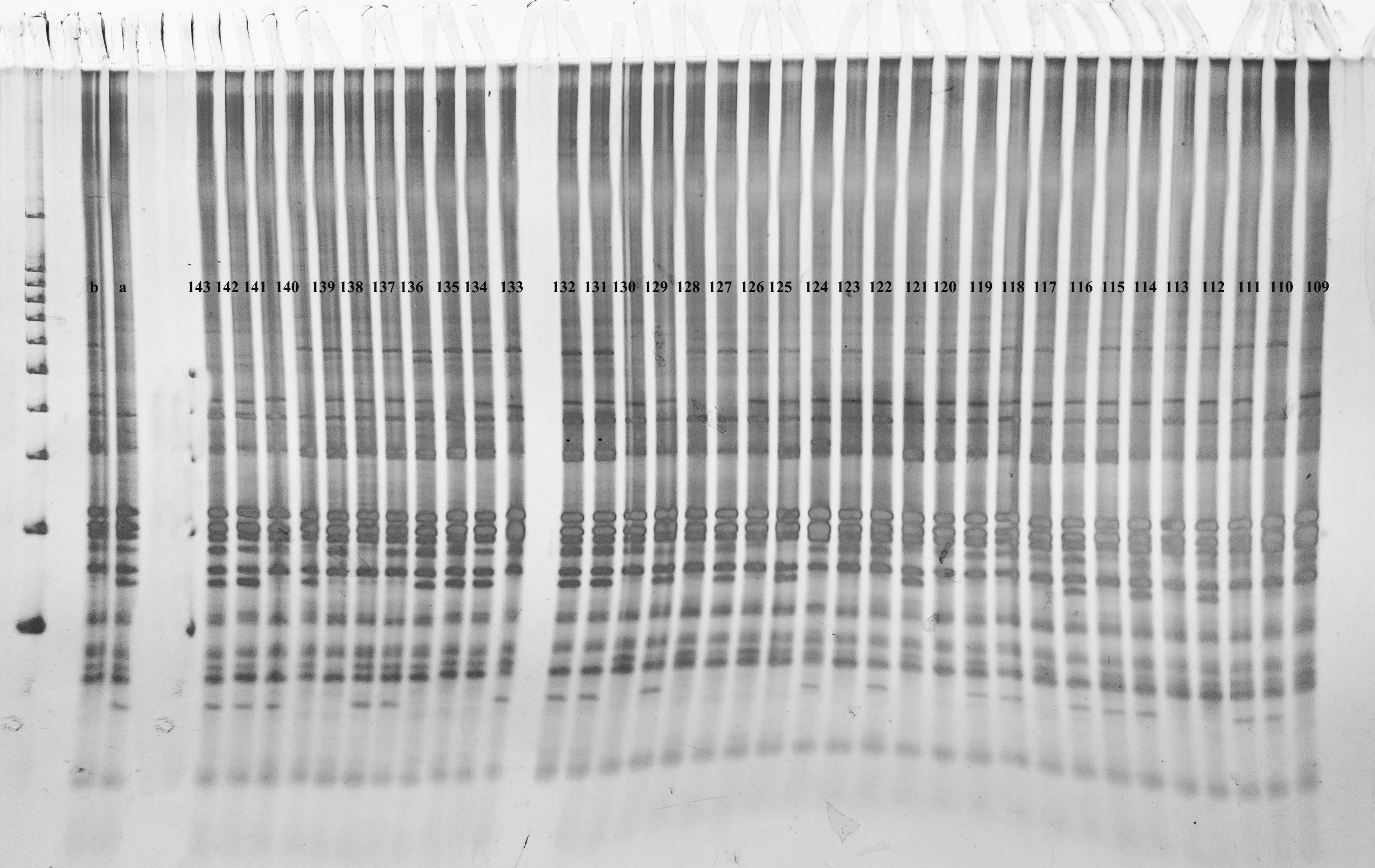

Supplement: Supplemental Information 8 — The compressed file name is the primer name, the electrophoresis lane number is expressed by the subfile name, and the lane number is marked above the electrophoresis lane in the picture. [file peerj-10-14442-s008.zip › BC1/44262/109-143+a,b(44262).jpg]

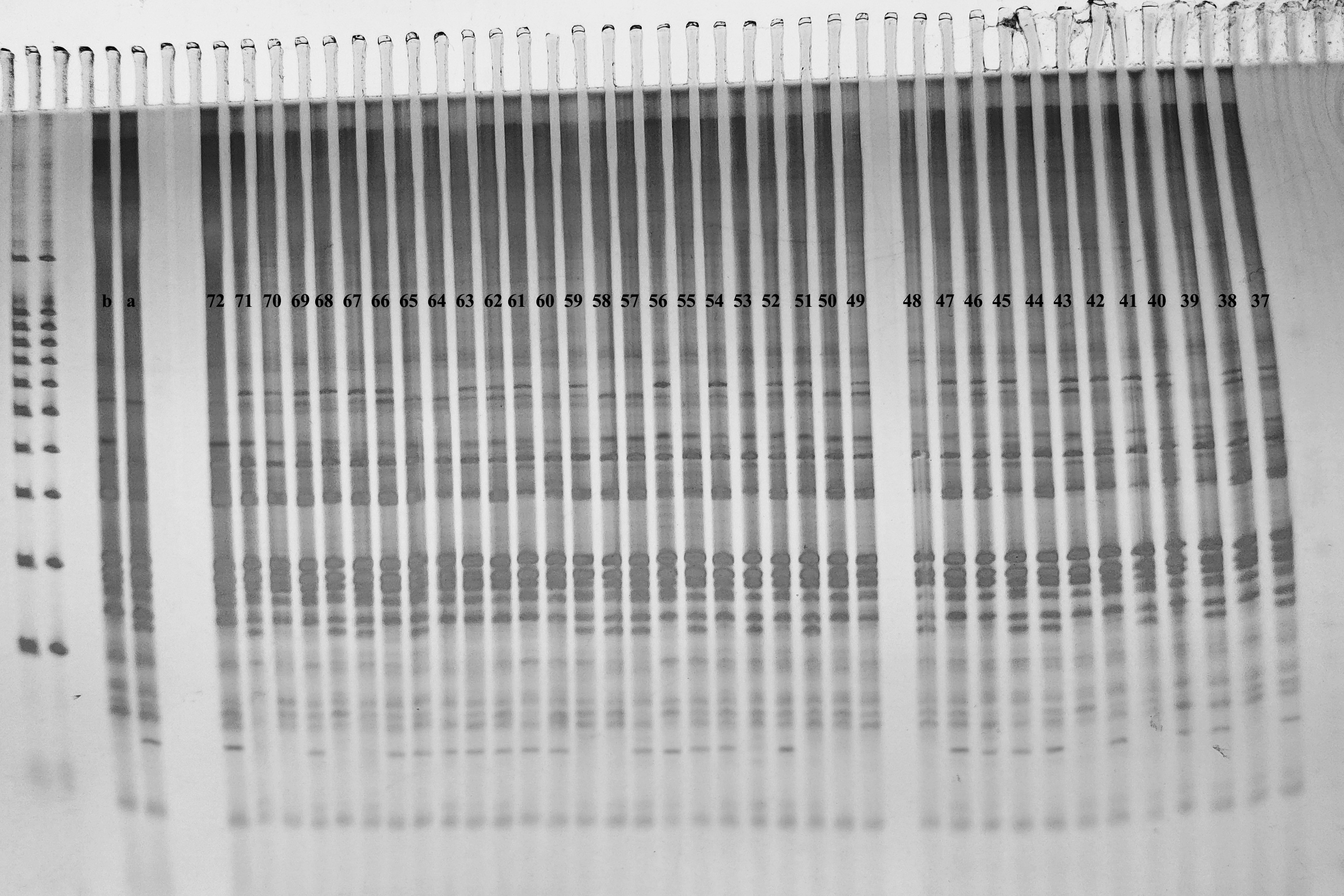

Supplement: Supplemental Information 8 — The compressed file name is the primer name, the electrophoresis lane number is expressed by the subfile name, and the lane number is marked above the electrophoresis lane in the picture. [file peerj-10-14442-s008.zip › BC1/44262/37-72+a,b(44262).jpg]

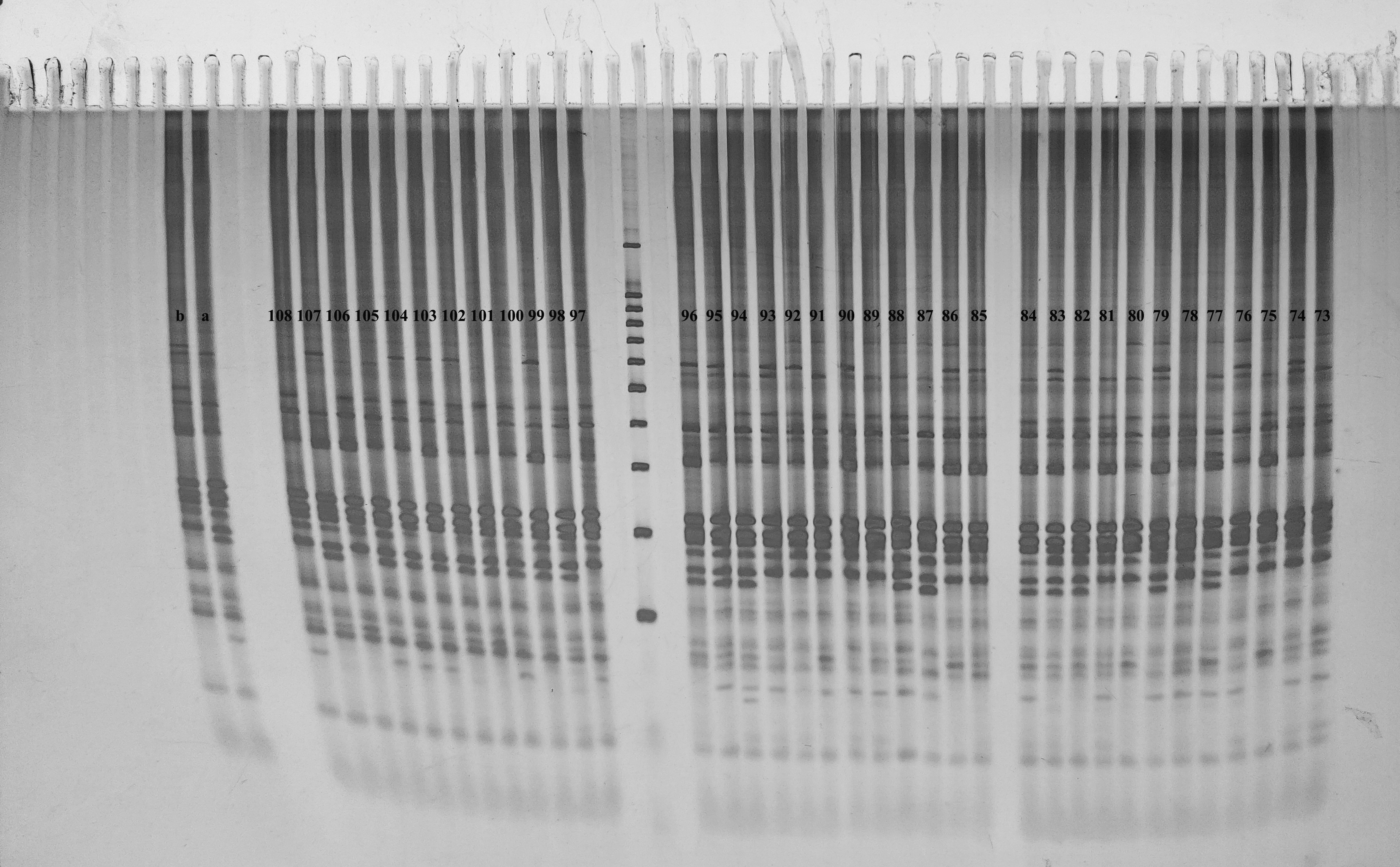

Supplement: Supplemental Information 8 — The compressed file name is the primer name, the electrophoresis lane number is expressed by the subfile name, and the lane number is marked above the electrophoresis lane in the picture. [file peerj-10-14442-s008.zip › BC1/44262/73-108+a,b(44262).jpg]

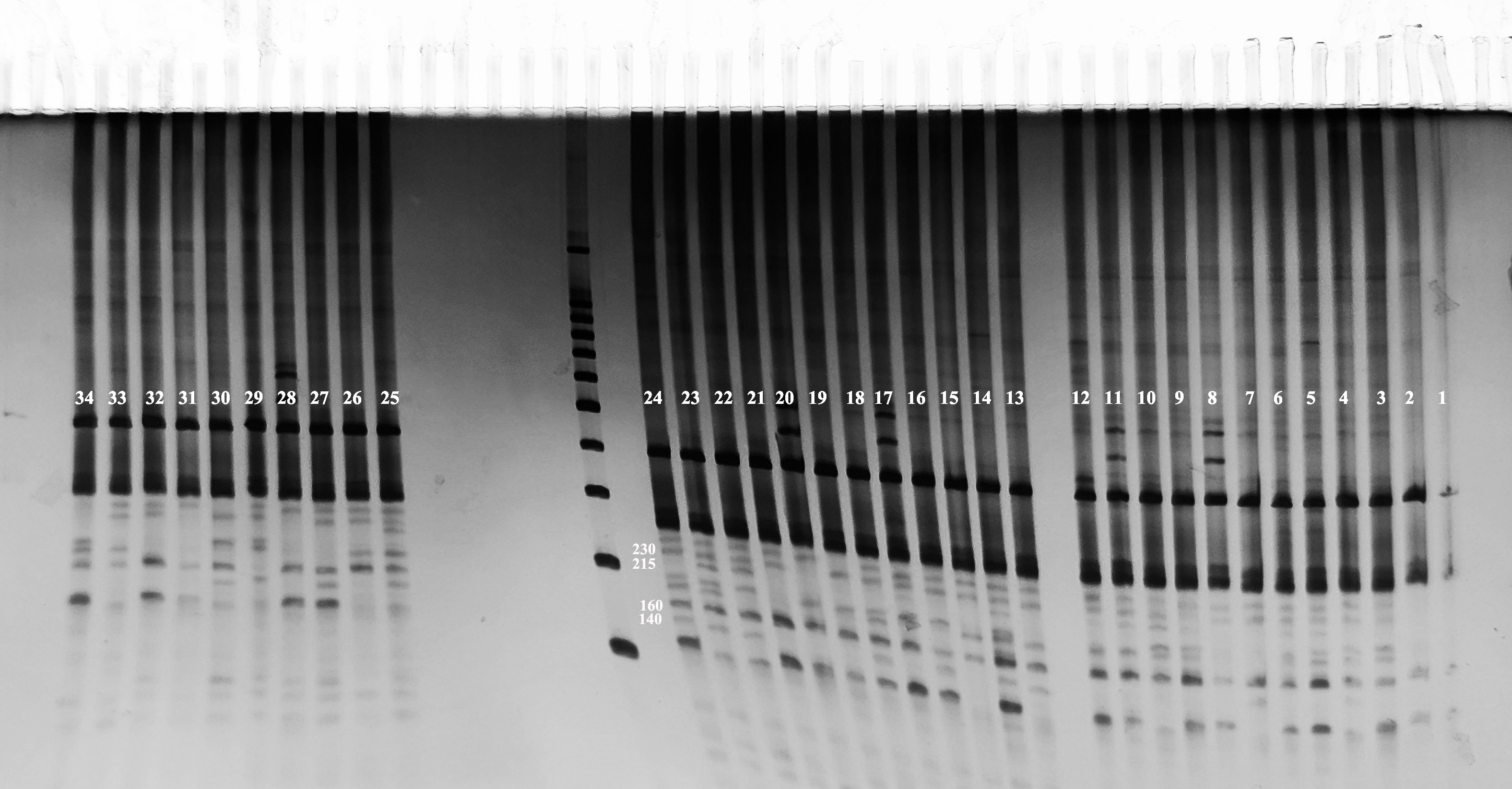

Supplement: Supplemental Information 8 — The compressed file name is the primer name, the electrophoresis lane number is expressed by the subfile name, and the lane number is marked above the electrophoresis lane in the picture. [file peerj-10-14442-s008.zip › BC1/60437/1-34(60437).jpg]

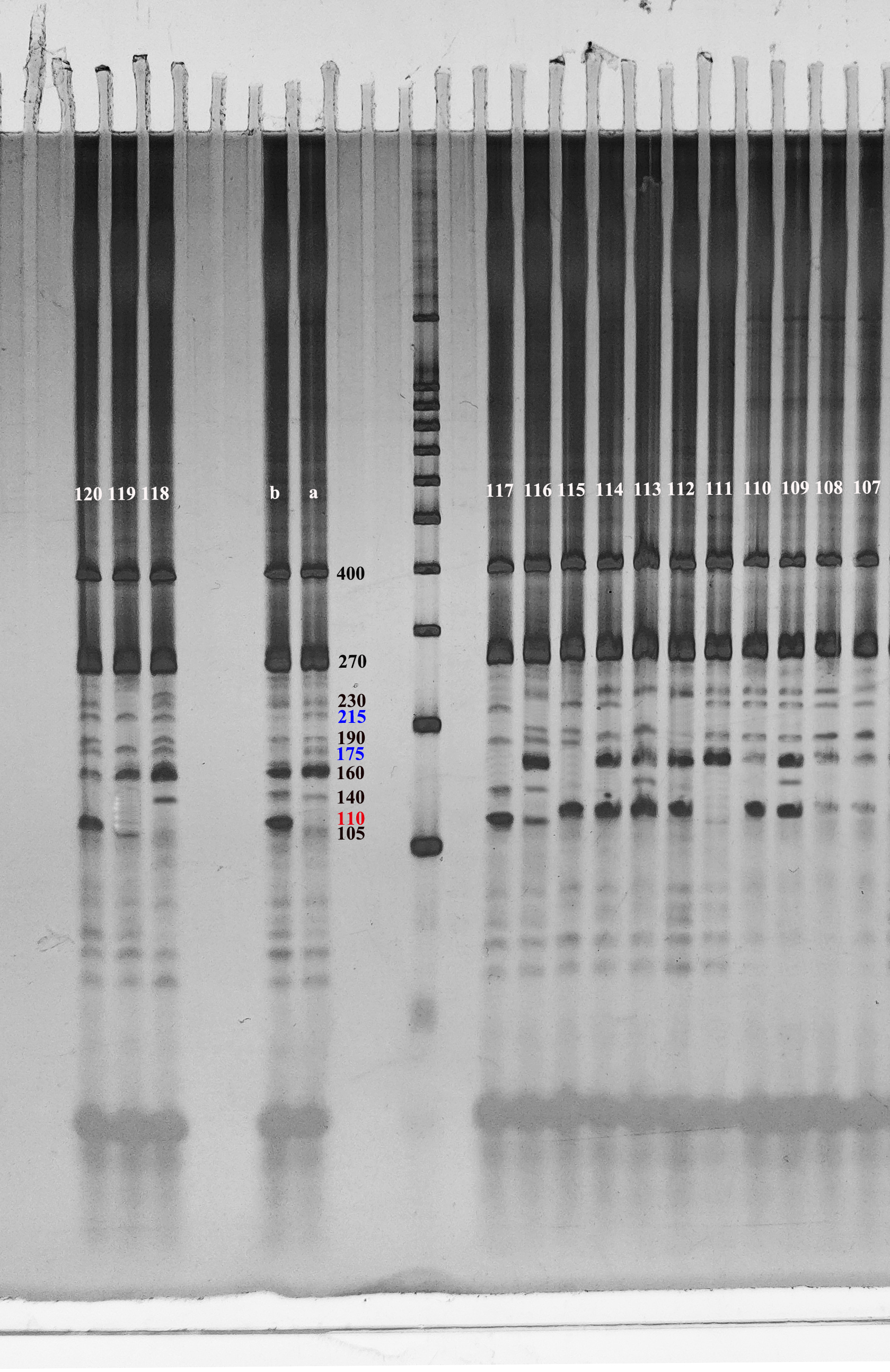

Supplement: Supplemental Information 8 — The compressed file name is the primer name, the electrophoresis lane number is expressed by the subfile name, and the lane number is marked above the electrophoresis lane in the picture. [file peerj-10-14442-s008.zip › BC1/60437/107-120+a,b(60437).jpg]

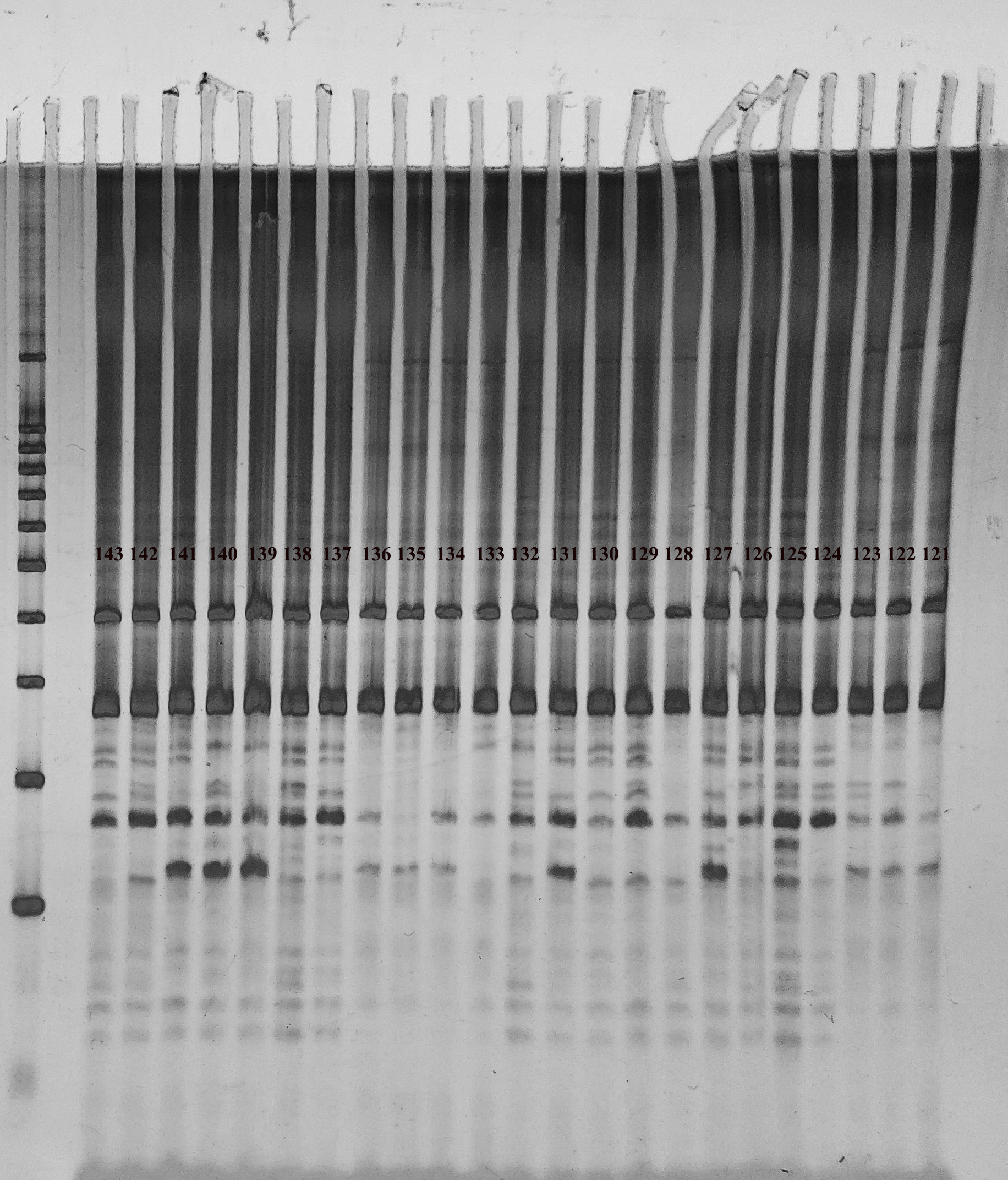

Supplement: Supplemental Information 8 — The compressed file name is the primer name, the electrophoresis lane number is expressed by the subfile name, and the lane number is marked above the electrophoresis lane in the picture. [file peerj-10-14442-s008.zip › BC1/60437/121-143(60437).jpg]

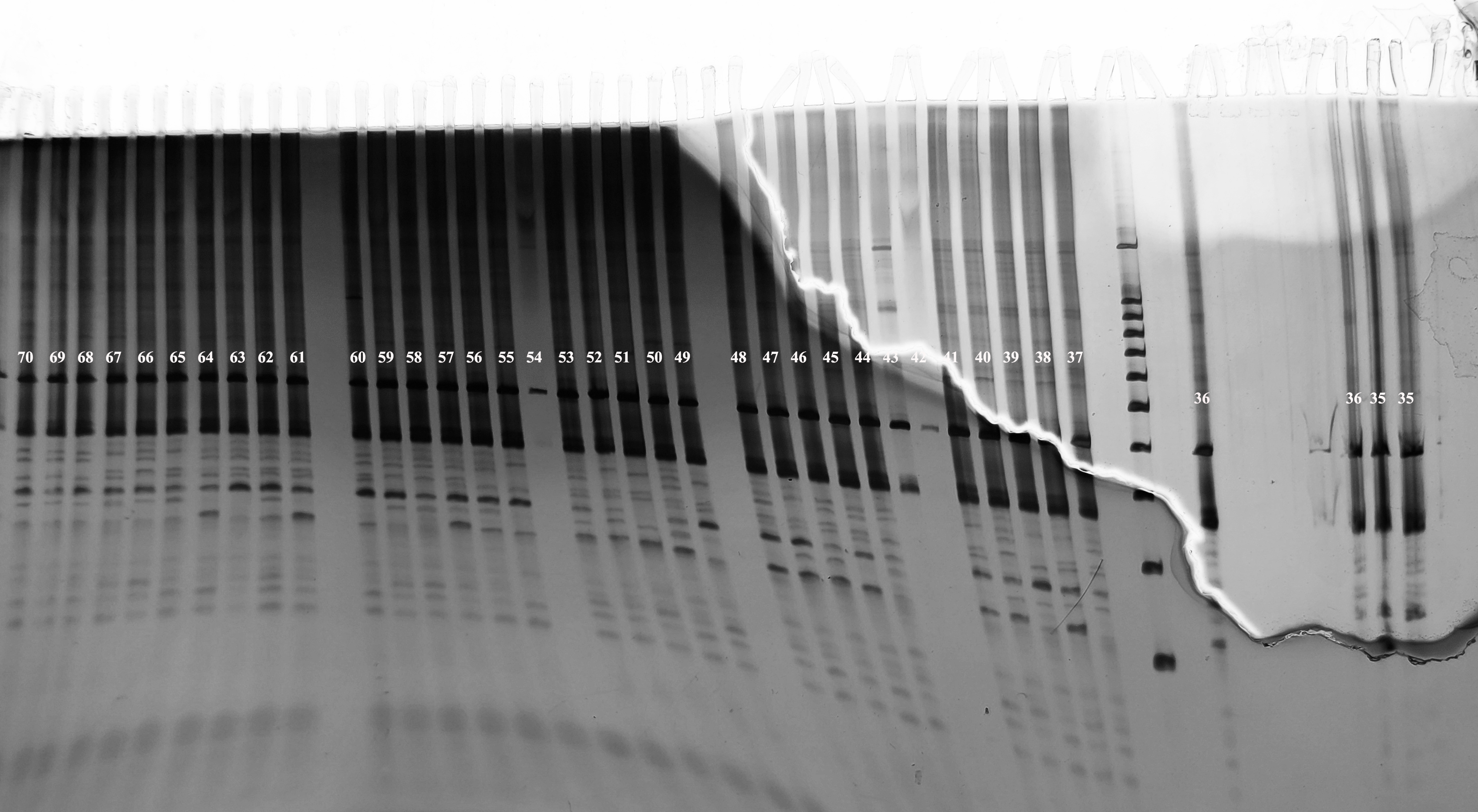

Supplement: Supplemental Information 8 — The compressed file name is the primer name, the electrophoresis lane number is expressed by the subfile name, and the lane number is marked above the electrophoresis lane in the picture. [file peerj-10-14442-s008.zip › BC1/60437/35-70(60437).jpg]

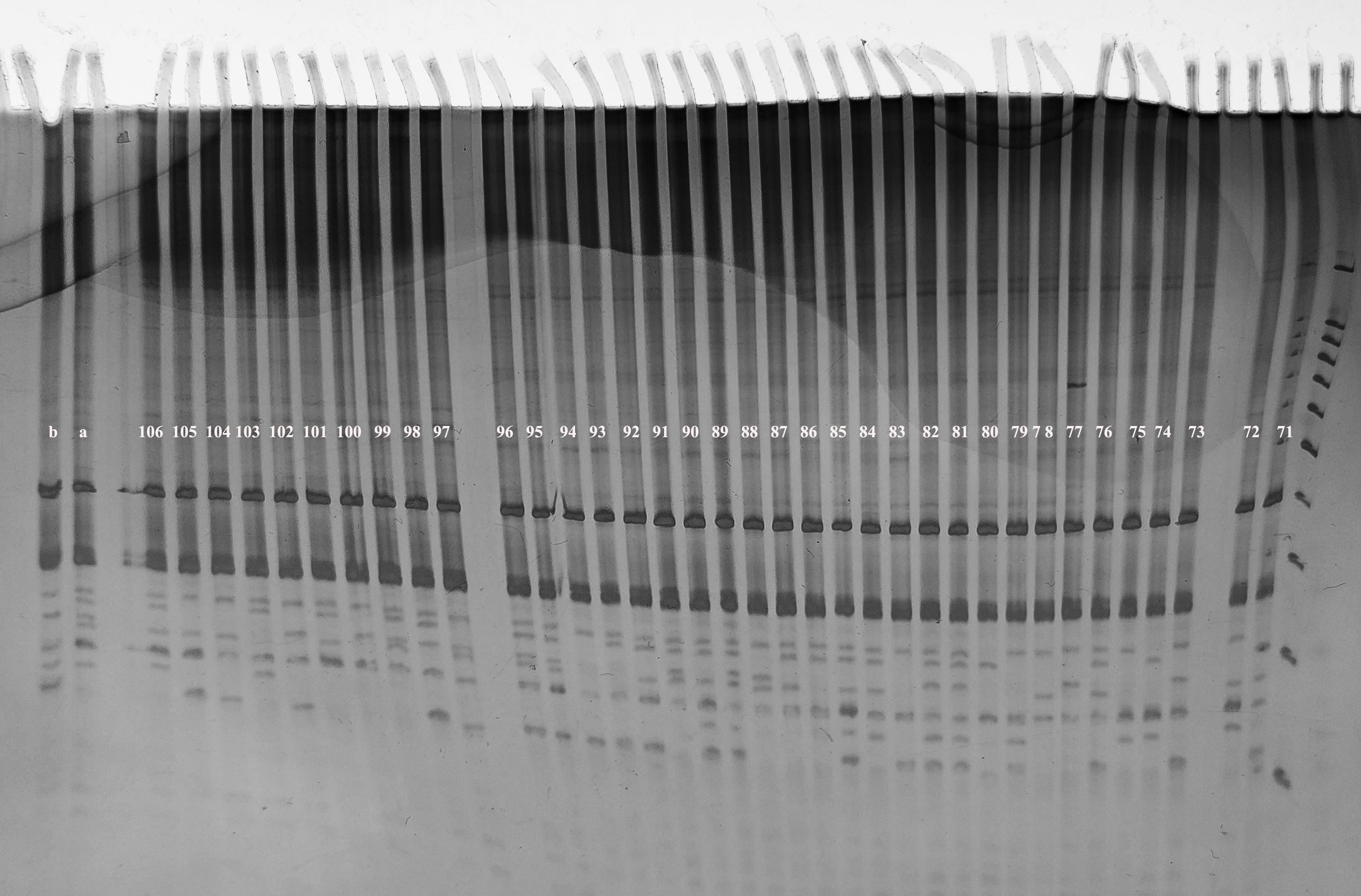

Supplement: Supplemental Information 8 — The compressed file name is the primer name, the electrophoresis lane number is expressed by the subfile name, and the lane number is marked above the electrophoresis lane in the picture. [file peerj-10-14442-s008.zip › BC1/60437/71-106+a,b(60437).jpg]

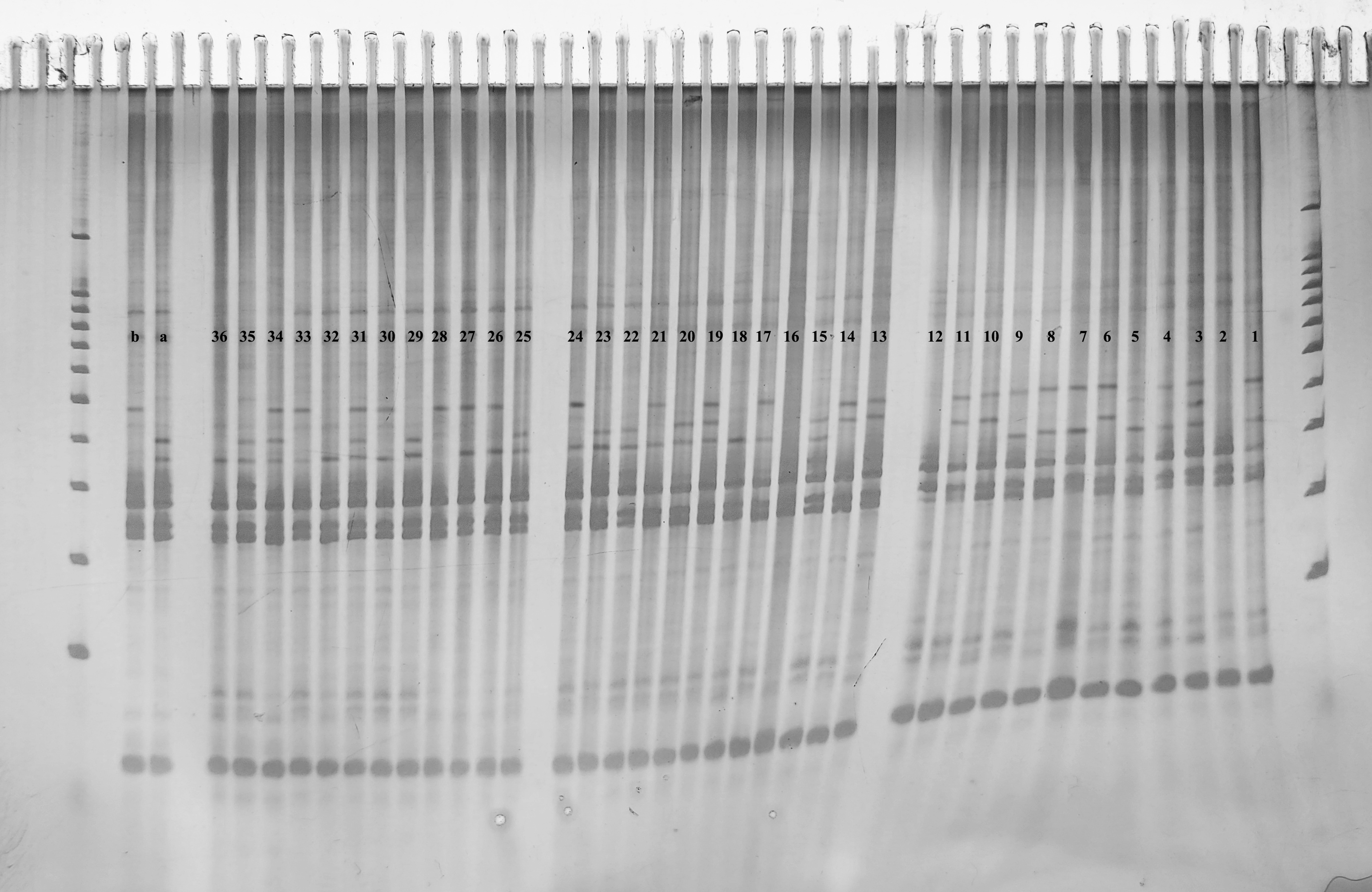

Supplement: Supplemental Information 8 — The compressed file name is the primer name, the electrophoresis lane number is expressed by the subfile name, and the lane number is marked above the electrophoresis lane in the picture. [file peerj-10-14442-s008.zip › BC1/6181A/1-36+a,b(6181A).jpg]

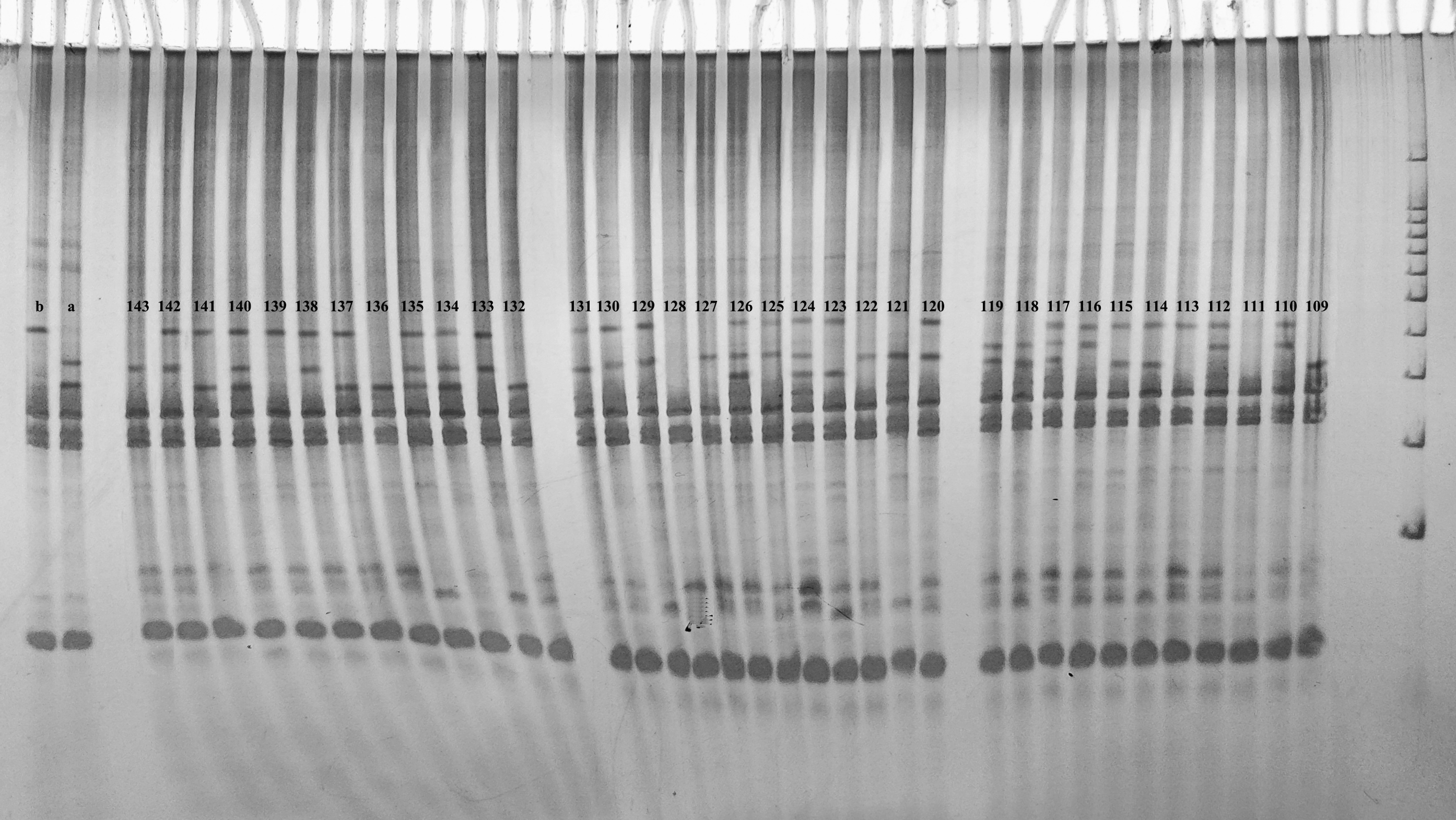

Supplement: Supplemental Information 8 — The compressed file name is the primer name, the electrophoresis lane number is expressed by the subfile name, and the lane number is marked above the electrophoresis lane in the picture. [file peerj-10-14442-s008.zip › BC1/6181A/109-143+a,b.jpg]

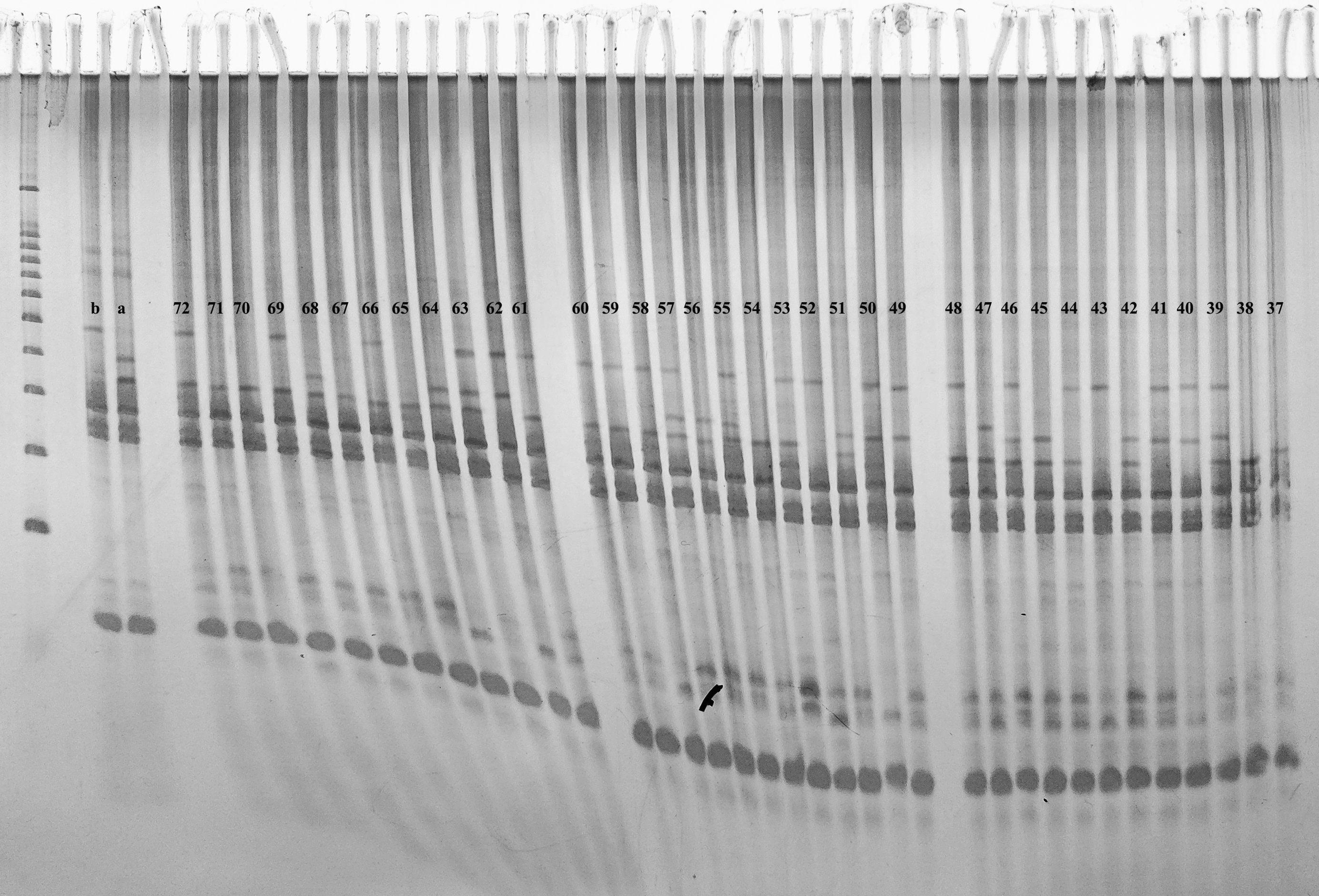

Supplement: Supplemental Information 8 — The compressed file name is the primer name, the electrophoresis lane number is expressed by the subfile name, and the lane number is marked above the electrophoresis lane in the picture. [file peerj-10-14442-s008.zip › BC1/6181A/37-72+a,b(6181A).jpg]

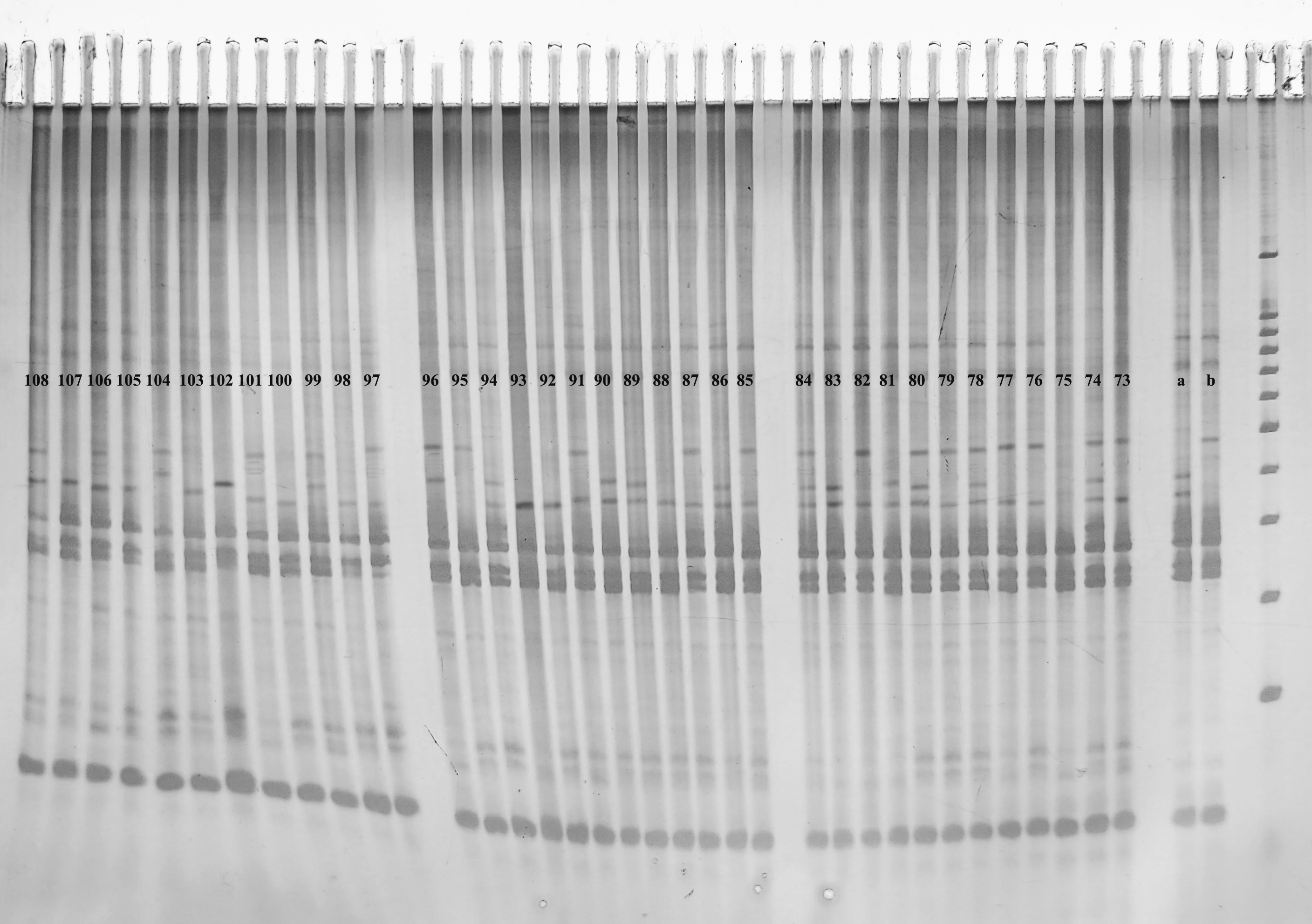

Supplement: Supplemental Information 8 — The compressed file name is the primer name, the electrophoresis lane number is expressed by the subfile name, and the lane number is marked above the electrophoresis lane in the picture. [file peerj-10-14442-s008.zip › BC1/6181A/73-108+a,b(6181A).jpg]
