# Supplementary material for: Hybrid purity identification using EST-SSR markers and heterosis analysis of quantitative traits of Russian wildrye
Source: PeerJ. 2022 Nov 30;10:e14442. doi: 10.7717/peerj.14442 (PMC9744169; doi:10.7717/peerj.14442)
Supplement: Supplemental Information 10 [file peerj-10-14442-s010.zip › raw-code & result/R-correlation analysis-raw code.docx]

install.packages("see")

install.packages("correlation")

library(see)

library(correlation)

getwd()

df<-read.csv("R-correlation analysis-raw data.csv")

df

correlation(df[,2:14])->df.cor

df.cor

write.table(df.cor,file="R-correlation analysis results.csv",quote = F,row.names = F)

plot(summary(df.cor),show_values=T,show_p=T,size_point=1.4)
