# Supplementary material for: Hybrid purity identification using EST-SSR markers and heterosis analysis of quantitative traits of Russian wildrye
Source: PeerJ. 2022 Nov 30;10:e14442. doi: 10.7717/peerj.14442 (PMC9744169; doi:10.7717/peerj.14442)
Supplement: Supplemental Information 11 [file peerj-10-14442-s011.docx]

**data** zhucf;

input x1-x12;

cards;

123.33 181.33 281.50 29.67 0.27 11.50 0.60 39.50 106.10 0.27 2.54 19.72

137.17 87.33 349.33 27.16 0.41 13.37 0.58 33.70 109.31 0.39 3.54 9.68

131.00 153.67 170.67 21.80 0.39 11.76 0.79 36.50 179.25 0.46 2.57 28.21

143.00 173.33 299.67 38.78 0.48 6.91 0.76 27.60 71.48 0.18 2.50 12.44

135.00 138.67 143.67 22.30 0.30 10.37 0.56 34.70 122.12 0.31 2.54 16.96

104.33 8.00 160.33 24.08 0.37 8.89 0.51 26.35 58.68 0.15 2.57 0.43

125.50 99.67 184.00 27.10 0.29 13.65 0.78 37.90 130.48 0.41 3.09 13.21

139.33 97.00 197.50 23.33 0.34 12.31 0.53 34.60 88.04 0.23 2.57 8.97

131.50 68.00 187.33 23.83 0.33 11.53 0.65 36.00 140.67 0.47 3.30 9.50

128.75 37.50 252.00 24.48 0.39 11.22 0.57 30.00 75.42 0.25 3.37 2.71

128.00 45.00 102.00 24.95 0.34 10.44 0.62 31.30 97.12 0.22 2.27 3.60

123.25 79.00 180.00 22.93 0.35 9.67 0.54 27.30 108.87 0.24 2.27 8.07

132.67 97.00 238.67 23.98 0.36 10.43 0.44 30.00 71.14 0.24 3.37 7.03

124.50 49.00 252.00 25.70 0.35 12.00 0.79 34.50 88.81 0.31 3.44 4.29

119.92 70.50 222.67 19.97 0.34 11.40 0.61 36.40 137.84 0.42 3.04 9.67

115.77 76.00 200.67 21.17 0.32 10.01 0.68 27.00 74.13 0.23 3.07 5.38

99.17 9.50 125.33 12.77 0.24 7.88 0.59 23.70 43.38 0.08 1.60 0.41

119.33 18.00 158.67 20.98 0.39 10.61 0.59 32.00 94.06 0.29 3.05 1.74

115.50 31.00 114.00 16.58 0.25 8.44 0.48 26.70 49.45 0.15 3.12 1.46

122.25 91.50 215.67 30.13 0.35 8.57 0.59 30.00 80.78 0.18 2.35 8.20

126.17 60.00 176.33 20.15 0.32 7.13 0.58 25.70 97.51 0.27 2.82 5.92

112.00 59.50 189.33 17.51 0.38 9.33 0.49 28.00 85.59 0.22 2.70 5.16

108.50 39.00 149.50 20.80 0.30 8.72 0.60 25.60 69.14 0.18 2.62 2.60

122.83 77.50 197.33 20.03 0.35 11.25 0.53 35.10 82.13 0.24 2.97 6.96

120.50 64.00 120.00 22.57 0.34 10.18 0.65 29.60 74.32 0.24 3.27 4.46

109.50 2.00 73.33 18.80 0.36 8.77 0.50 26.63 34.60 0.09 2.60 0.07

127.33 66.50 170.00 18.93 0.35 10.14 0.69 31.10 63.85 0.20 3.12 3.13

116.50 23.67 237.67 24.03 0.43 10.27 0.56 29.00 77.98 0.26 3.40 1.84

102.92 30.00 204.67 26.90 0.27 8.83 0.54 23.00 30.73 0.06 1.92 0.71

102.00 20.00 142.33 26.60 0.33 10.93 0.50 27.27 80.25 0.23 2.82 1.31

106.42 29.00 147.67 20.72 0.30 9.88 0.60 33.00 66.65 0.20 3.02 1.78

105.13 22.00 99.67 19.73 0.39 9.68 0.63 25.80 51.02 0.09 1.85 0.69

114.33 102.67 154.00 21.83 0.33 8.83 0.64 29.30 59.16 0.17 2.90 6.20

106.50 19.33 107.00 17.10 0.29 10.43 0.60 31.50 89.08 0.24 2.67 1.77

123.83 49.83 130.33 17.92 0.34 11.99 0.52 36.30 158.19 0.48 3.00 7.61

129.67 66.00 138.67 27.18 0.35 12.96 0.57 32.50 85.43 0.25 2.95 5.16

116.67 18.00 202.00 24.38 0.36 7.87 0.53 27.10 55.55 0.14 2.52 1.00

120.67 25.00 207.33 22.58 0.43 10.23 0.62 27.10 62.04 0.17 2.80 1.46

84.50 8.50 181.67 16.58 0.34 8.83 0.47 26.28 50.97 0.11 2.15 0.42

106.33 27.33 188.33 18.83 0.26 9.73 0.62 33.50 84.60 0.24 2.90 2.48

122.83 128.00 188.33 22.33 0.26 10.94 0.60 32.60 72.45 0.19 2.67 9.73

106.50 32.67 164.67 19.88 0.22 8.73 0.47 29.20 67.79 0.21 3.05 2.17

119.83 46.33 184.67 23.83 0.43 10.23 0.50 32.00 64.85 0.19 2.97 3.07

122.00 50.50 180.00 16.98 0.34 10.09 0.64 29.90 77.69 0.31 3.95 4.10

98.83 22.33 164.67 20.53 0.36 8.98 0.58 26.00 60.33 0.17 2.87 1.36

111.17 40.00 131.00 17.58 0.37 9.99 0.52 25.00 60.94 0.19 3.12 2.46

131.00 77.00 194.00 22.43 0.38 10.57 0.52 33.00 82.87 0.26 3.14 6.50

115.93 30.00 212.00 21.18 0.31 10.56 0.57 24.80 68.42 0.14 2.10 2.07

138.33 88.33 178.50 23.23 0.44 8.70 0.66 30.70 29.83 0.10 3.27 2.59

140.50 85.00 153.50 26.38 0.34 11.95 0.69 35.00 49.67 0.16 3.24 4.32

105.50 23.50 118.33 21.03 0.40 8.32 0.68 22.70 45.84 0.12 2.52 0.98

94.17 14.33 154.00 21.93 0.30 8.33 0.52 24.95 70.21 0.14 1.97 1.11

137.83 85.67 113.00 23.28 0.40 10.56 0.60 35.30 77.70 0.20 2.62 6.70

128.33 42.67 220.50 24.17 0.34 10.36 0.64 29.30 84.38 0.27 3.25 3.55

123.83 101.00 135.50 26.98 0.33 10.24 0.49 34.30 42.29 0.13 3.05 5.34

123.33 51.50 149.50 18.53 0.26 9.66 0.69 32.40 86.05 0.27 3.20 4.48

73.17 3.50 93.50 15.57 0.32 7.62 0.56 25.00 32.44 0.08 2.17 0.12

127.50 44.50 189.00 26.12 0.36 11.63 0.68 34.00 62.06 0.14 2.35 2.76

110.17 62.50 169.00 32.12 0.42 9.59 0.59 28.53 33.46 0.06 1.82 1.51

95.67 32.00 203.00 16.48 0.32 7.21 0.66 20.00 54.09 0.14 2.47 1.55

113.17 30.00 279.00 22.37 0.35 9.56 0.72 28.00 77.90 0.27 3.47 2.43

143.50 96.67 156.00 23.08 0.40 12.06 0.72 33.60 68.35 0.22 3.27 6.85

118.00 52.33 212.50 19.62 0.36 10.32 0.59 32.40 86.03 0.29 3.35 4.34

116.83 37.50 162.00 23.82 0.39 10.40 0.57 26.10 59.51 0.20 3.42 1.89

129.17 90.67 121.50 19.00 0.31 11.61 0.63 42.40 120.46 0.31 2.54 10.91

125.50 133.50 110.50 21.72 0.37 9.86 0.63 32.50 51.69 0.17 3.07 7.56

123.50 50.33 185.50 26.38 0.49 11.79 0.71 33.20 103.03 0.34 3.67 5.17

121.33 23.00 158.00 26.72 0.34 10.59 0.53 33.20 73.54 0.21 2.87 1.72

131.50 71.00 158.00 20.77 0.40 12.04 0.53 31.40 92.75 0.30 3.24 6.71

119.50 76.00 166.50 27.75 0.38 12.03 0.51 31.80 31.92 0.10 3.02 1.66

124.83 65.00 173.50 28.43 0.35 10.23 0.61 28.60 66.46 0.17 2.60 3.72

138.33 93.00 181.50 19.65 0.32 10.20 0.63 34.70 25.49 0.07 2.67 2.39

135.50 59.50 180.50 27.35 0.32 11.12 0.59 35.50 87.81 0.32 3.65 5.73

136.50 78.33 269.00 29.65 0.37 13.68 0.60 35.40 47.21 0.11 2.37 3.97

114.83 26.00 181.00 23.07 0.34 10.51 0.69 25.20 56.62 0.18 3.25 1.28

134.17 56.00 207.00 18.60 0.36 11.34 0.48 31.20 47.86 0.16 3.37 2.90

117.83 41.00 204.00 25.70 0.34 10.45 0.56 31.00 54.16 0.15 2.85 2.21

123.17 34.00 182.00 19.02 0.32 12.61 0.52 34.10 83.04 0.22 2.65 2.97

135.50 78.00 87.00 25.53 0.31 11.56 0.49 30.50 48.46 0.16 3.32 2.97

114.17 51.50 177.50 25.02 0.37 9.41 0.43 26.20 41.35 0.10 2.37 2.06

101.83 45.50 135.00 23.10 0.46 5.78 0.65 17.00 42.44 0.11 2.64 1.79

140.83 128.00 187.00 25.80 0.60 11.47 0.48 34.30 84.57 0.23 2.70 10.69

132.17 95.50 242.00 21.57 0.31 10.23 0.56 33.00 39.96 0.12 2.87 3.46

127.50 70.00 215.00 27.27 0.35 11.80 0.58 33.00 69.87 0.23 3.31 4.78

125.17 87.00 214.00 15.42 0.37 8.26 0.78 28.60 69.58 0.22 3.20 6.69

125.00 61.00 170.00 20.90 0.31 9.47 0.61 29.60 39.60 0.14 3.42 2.51

141.50 81.50 170.50 21.63 0.37 9.60 0.56 31.30 32.03 0.06 1.94 2.48

135.17 68.00 175.50 24.53 0.32 11.92 0.61 32.70 88.14 0.23 2.62 5.98

118.17 45.33 135.00 23.90 0.24 10.55 0.61 30.00 67.45 0.20 3.02 3.11

127.83 61.33 190.00 18.00 0.30 10.61 0.64 35.00 90.90 0.28 3.12 6.12

120.17 78.33 131.50 25.18 0.35 10.91 0.65 31.50 86.75 0.26 2.91 6.92

140.17 130.83 169.50 22.87 0.30 10.70 0.59 29.00 39.13 0.12 3.17 5.29

125.83 89.33 155.00 18.46 0.31 7.83 0.52 33.00 67.54 0.19 2.80 6.83

123.67 41.33 212.50 19.03 0.34 11.43 0.69 32.00 80.53 0.27 3.34 3.61

110.50 14.33 172.50 26.42 0.34 12.44 0.53 32.00 69.71 0.24 3.39 1.04

125.83 68.33 101.50 21.83 0.26 8.85 0.69 29.10 51.96 0.20 3.82 3.06

126.33 257.33 79.00 18.78 0.43 11.33 0.69 41.30 141.89 0.38 2.65 35.62

132.67 175.33 152.50 19.58 0.34 11.61 0.70 35.50 92.92 0.29 3.10 16.52

122.17 47.83 136.50 21.00 0.39 7.55 0.53 25.00 51.53 0.15 2.97 2.14

120.00 63.83 178.50 20.88 0.40 10.81 0.65 34.50 98.81 0.28 2.85 6.36

134.43 35.33 157.50 23.65 0.31 10.78 0.63 29.40 82.61 0.24 2.90 3.01

116.83 56.83 122.00 19.52 0.36 11.99 0.69 35.30 73.70 0.22 3.00 4.77

123.60 84.33 159.00 21.33 0.27 11.89 0.49 35.30 78.04 0.21 2.70 6.50

115.23 99.33 166.00 21.48 0.30 10.83 0.47 34.10 49.23 0.15 3.07 5.08

110.67 76.33 85.50 24.18 0.37 9.11 0.53 28.00 73.66 0.21 2.85 5.80

128.83 80.83 160.50 24.12 0.35 9.43 0.54 30.90 35.43 0.12 3.45 2.86

117.33 85.83 159.00 20.13 0.26 11.65 0.61 32.60 119.50 0.28 2.35 10.29

128.67 70.33 124.00 24.57 0.42 9.55 0.53 33.00 37.90 0.11 2.87 2.62

131.33 67.33 136.00 22.53 0.39 13.88 0.56 35.20 110.34 0.35 3.21 7.53

；

**proc** **print**;

**run**;

**proc** **princomp** data = zhucf n = **9** out = w1 outstat = w2;

var x1-x12;**proc** **print** data = w1;

**run**;
